# The Essential Network (TEN): consulting stakeholders and experts to better understand implementation of a blended care mental health support services for Australian health professionals

**DOI:** 10.1080/00049530.2024.2425614

**Published:** 2024-11-13

**Authors:** Matthew Coleshill, Kelby Fransisca, Xiaoling Du, Melissa Black, Jill M. Newby, Samuel Harvey, Helen Christensen, Peter Baldwin

**Affiliations:** a Black Dog Institute, Sydney, Australia; b UNSW Medicine & Health, University of New South Wales, Sydney, Australia; c School of Psychological Sciences, Macquarie University, Sydney, Australia; d School of Psychology, University of New South Wales, Sydney, Australia

**Keywords:** Blended care, mental health, qualitative, health professionals, healthcare workers, COVID-19

## Abstract

**Objective:**

The Essential Network (TEN) is a blended care mental health support service for Australian health professionals. We conducted a series of semi-structured interviews with key stakeholders and researchers to understand health professionals’ needs, canvas suggested changes to TEN, and examine methods of improving service uptake.

**Method:**

Nine semi-structured individual or group interviews were conducted with 10 TEN stakeholders (external stakeholders) and eight interviews were conducted with 18 researchers or related roles with experience implementing or evaluating mental health services for health professionals (internal experts). De-identified transcripts were thematically analysed using an inductive and deductive approach.

**Results:**

Participants highlighted the need for confidentiality, with mandatory reporting concerns being a key barrier to health professionals engaging with mental health services. External stakeholders viewed digital services as advantageous due to accessibility and anonymity, although both groups noted that concerns around effectiveness were a barrier to engagement with digital services. Both groups agreed that peer endorsement was key to implementation.

**Conclusions:**

Digital services were viewed as promising, but best employed alongside person-to-person options in a blended care format. Services that address the unique workplace culture of healthcare, including stigma and systemic barriers to help-seeking, can create effective and scalable support for health professionals.

## Key Points

What is already known about this topic:

(1) Health professionals are at a greater risk of poor mental health compared to the general public.

(2) The COVID-19 pandemic has placed further strain upon health professionals, highlighting the need for more tailored mental health services.

(3) Cultural and systemic issues exist within healthcare that act as barriers to help-seeking.

What this topic adds:

(1) Digital services were viewed as best employed alongside person-to-person services in a blended care format.

(2) In addition to already established barriers, the volume of information health professionals receive may have obscured information about relevant support services.

(3) Peer endorsement was seen as crucial to effectively driving the usage of mental health support services for health professionals.

## Introduction

Health professionals are well-established as an occupational group at risk of poor mental health (Wallace et al., 2009). This risk, exacerbated by health professionals’ workplace culture and a lack of help-seeking (Edwards & Crisp, 2017; Muhamad Ramzi et al., 2021), is compounded by the impact of the COVID-19 pandemic (Greenberg et al., 2020). While ongoing, the consequences of the COVID-19 pandemic on health professionals are clear, with high rates of post-traumatic stress disorder (PTSD) (49%), anxiety (40%), and depression (37%) being apparent (Saragih et al., 2021). To address known barriers to health professionals accessing mental health services, particularly stigma, confidentiality, and mandatory reporting, health professionals require tailored, evidence-based mental health services specific to their needs, workplace culture, and experiences. The Essential Network (TEN) is one such mental health support service developed by the Black Dog Institute (BDI) and funded by the Australian Federal Department of Health as part of their national COVID-19 response strategy to support the mental health of health professionals through the pandemic (Baldwin et al., 2022).

During the development of TEN in 2020, Australian peak professional bodies representing health professionals and mental health organisations were assembled as stakeholders to ensure the service was meeting the needs of health professionals. The resulting service was an integrated blended care service that provided educational resources, self-guided digital treatment programs, digital mental health assessments alongside free person-to-person consultations available in person or via telehealth through BDI Clinical Service (Baldwin et al., 2022).

Following the rapid development and launch of TEN, we conducted a series of semi-structured interviews with TEN stakeholders to 1) understand the needs of health professionals from the perspective of each stakeholder; 2) canvas suggestions for changes to TEN to better address health professionals’ mental health needs; and 3) examine methods of implementing TEN to improve uptake among health professionals as part of a wider evaluation (Coleshill et al., 2022). In addition to interviewing external stakeholders, we have also conducted a series of internal semi-structured interviews with researchers and related roles at BDI who had experience with health professionals’ mental health or service implementation to further understand implementing mental health services in healthcare. The present study describes the qualitative analysis of these interviews.

## Methods

### Study design

One-hour semi-structured interviews with individuals or small groups were conducted by video conferencing (e.g., Microsoft Teams, Zoom). Participants were invited to participate by email and provided with an online participation information sheet and consent form. Each participant was provided with a $135 gift card as reimbursement for their time.

### Participants

Participants were recruited from a TEN external stakeholder group or internally from BDI staff. TEN external stakeholders (external stakeholders) representing peak health professional bodies or mental health organisations were approached to participate in the study. The study aimed to recruit between 10–15 external stakeholders and 15–20 internal experts. This sample size was deemed sufficient to answer the research questions based on the concept of information power, wherein recruiting participants with experience highly relevant to the study’s aims provides an information-rich dataset (Malterud et al., 2016).

To comply with the Human Research Ethics Panel approval for the study, we are unable to name the professional bodies or organisations due to the risk of identifying participants as representatives of their respective organisations. BDI staff (internal experts) were eligible if they had experience of either 1) conducting research on health professionals’ mental health or implementing interventions or services for use by health professionals, or 2) if they had experience communicating or engaging with health professionals (i.e., promotion of mental health awareness among, or mental health services for, health professionals). Eligible BDI staff known to the authors were approached to participate in the study alongside snowballing recruitment, whereby other potentially eligible participants were suggested.

### Data collection

Two semi-structured interview guides were developed for each participant group – external stakeholders and internal experts (Supplementary Material 1). These interview guides were developed using the Consolidated Framework for Implementation Research (CFIR) (Damschroder et al., 2009), as well as the published literature on implementation in a healthcare setting (Breimaier et al., 2015; Keith et al., 2017) and health professionals’ mental health (Bianchi et al., 2016; Edwards & Crisp, 2017; Muhamad Ramzi et al., 2021; Zaman et al., 2022). Key topics included the mental health needs of health professionals (broadly or for specific professions, depending on the experience of participant), feedback on TEN, strategies to engage with health professionals, and ways of implementing TEN to improve the uptake of the service.

Interviews were conducted by MJC, MJB, and PAB between 30 June 2021 and 6 August 2021. Participants from the same TEN stakeholder organisation or BDI team were interviewed together in semi-structured group interviews. Interviews lasted on average 62 minutes (ranging from 42 to 87 minutes). Each interview was video recorded, transcribed using the video conferencing software, corrected manually where needed by MJC, KF, and XD, and deidentified.

### Data analysis

Transcripts were analysed using reflexive thematic analysis using both deductive and inductive approaches (Braun & Clarke, 2021). Prior to open coding, MJC used a deductive approach to generate a set of meta-themes. Meta-themes were deductively drawn from the CFIR (Damschroder et al., 2009), implementation research employing the CFIR (Breimaier et al., 2015; Keith et al., 2017), and research on the mental health of health professionals (Bianchi et al., 2016; Edwards & Crisp, 2017; Muhamad Ramzi et al., 2021; Zaman et al., 2022).

Subthemes were inductively identified from the transcribed interviews across several stages: data familiarisation wherein transcripts were read without coding; development of initial codes wherein commonalities and consistent patterns in participant narratives were identified through open coding using a constant comparative approach; aggregation of initial codes into potential subthemes; review of refinement of subthemes through discussion; and clear naming and defining of resulting subthemes (Braun & Clarke, 2021). Open coding was conducted independently by KF and XD, with MJC analysing a subset of interviews to ensure reliability of inductive analysis. Thematic analysis was performed separately for both the external stakeholder and internal expert participant groups, with the resulting themes synthesised where appropriate.

For personal reflexivity, KF was naïve to data and health professionals’ mental health needs and workplace culture. XD was naïve to data but had relevant professional experience that led to complementary insights during inductive coding. MJC was familiar with data and published literature on mental health needs and workplace culture which influenced initial codes and inductive coding.

### Ethics approval

The study was approved by the UNSW Human Research Ethics Panel (HREAP ID: 3500). All participants provided informed consent to participate in the study. All methods were carried out in accordance with the National Statement on Ethical Conduct in Human Research.

## Results

**Figure 1.**
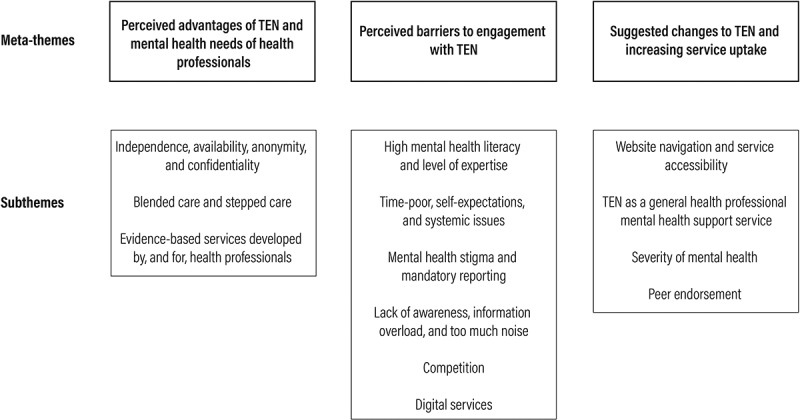
Graphical depiction of meta-themes deductively drawn from the literature and subthemes inductively identified through open coding.

A total of 10 external stakeholders participated in 9 interviews. Participants were mostly female (80%), with 6 (60%) representing peak Australian health professional organisations and 4 (40%) representing Australian mental health organisations. A total of 18 internal experts participated in 8 interviews. These participants were mostly female (88.9%), with 11 (61.1%) having experience of researching and/or implementing mental health interventions among health professionals, 4 (22.2%) engaging health professionals with mental health services through marketing, and 3 (16.7%) engaging health professionals through educational services. Figure 1 depicts meta-themes deductively drawn from the literature and subthemes inductively identified through open coding. Illustrative quotes from external stakeholders and internal experts supporting meta-themes and subthemes can be found in Supplementary Material 2.

## Perceived advantages of TEN and mental health needs of health professionals

### Independence, availability, anonymity, and confidentiality

TEN not being provided as part of Medicare or as part of an organisations’ Employee Assistance Program (EAP) was highlighted by as a key advantage due to health professionals needs around confidentiality. As health professionals are embedded within their local healthcare system, they do not feel comfortable engaging with other health professionals or services with whom they have professional relationships (Quote 1). This lack of confidentiality was seen as particularly important for health professionals in regional and remote Australia, where the limited number of services hindered accessing care discretely. The independence of TEN was also seen as a source of confidentiality compared to other services, particularly in regard to concerns around mandatory reporting and the potential impact that engaging with mental health services could have on health professionals’ registration and career progression (Quote 2). Indeed, the information provided by TEN on mandatory reporting was highlighted as a useful resource considering how hesitancy to engage with mental health services can culminate in poor health outcomes. Interestingly, discussions around confidentiality varied between different health professionals, with nurses being less concerned about mandatory reporting than doctors (Quote 3).

TEN being available to health professionals at no cost was seen as beneficial as it ensured health professionals unable to currently access Medicare are still able to receive mental health support without paying fees for private services (Quote 4). Indeed, TEN being freely available to health professionals was seen as necessary due to the substantial burden placed upon health professionals during the COVID-19 pandemic.

### Blended care and stepped care

External stakeholders saw blended care services as the gold standard for mental health support due to their perceived effectiveness, with digital components also being seen as part of a general trend in mental health services (Quote 5). One of the main perceived advantages of having a digital component was reducing barriers for health professionals to seek help (Quote 6).

Similarly, external stakeholders also highlighted TEN’s stepped care approach as an advantage, where health professionals can access varying levels of care based on their needs in a timely manner. This was particularly evident in more severe hypothetical situations, where TEN was seen as being able to quickly provide person-to-person mental health support akin to mental health crisis services (Quote 7). These views were linked to notions of continuity of care and TEN acting as a referral pathway into existing publicly funded mental health support services. In line with this, external stakeholders also pointed to how oversaturated the mental health space can be for health professionals (Quote 8), with TEN acting as a mental health hub to facilitate referral pathways.

### Evidence-based services developed by, and for, health professionals

In terms of health professionals’ needs, internal experts noted that health professionals required clear evidence supporting the effectiveness of services they engaged with (Quote 9). Understandably, this was not only due to health professionals being a highly educated group aware of research but also due to the emphasis on evidence and effectiveness in the treatments they provide to patients. Internal experts also frequently discussed the role of peers in health professionals’ mental health needs. An intervention being developed by-and-for health professionals was seen as important for ensuring that both content was relevant and good uptake (Quote 10). Indeed, peer support groups were pointed to as a reliable and valuable service that should form a part of mental health support services for health professionals (Quote 11).

## Perceived barriers to engagement with TEN

### High mental health literacy and level of expertise

Both groups highlighted several barriers to health professionals engaging with TEN, or the implementation of mental health services for health professionals broadly. A common theme was that, by the nature of their work, many health professionals have higher mental health literacy than the public, as well as expertise in managing patients’ mental health. This experience may make them more particular about the services that they engage with, especially person-to-person services. While it was noted by internal experts that health professionals understanding of evidence-based practice made them more agreeable to participating in research and evaluations (Quote 12), their knowledge was also seen as a barrier to engagement as health professionals may not see the value of an intervention or if the procedure differed from their preferred approach to care (Quote 13). Further, there was a view that doctors may find less value in a service where they see themselves as having a higher level of understanding than the practitioner (Quote 15). Perceived expertise of a mental health professional (or lack thereof) as a barrier to help-seeking was linked to views of a medical hierarchy, wherein medical professionals often want to be treated by someone of equivalent or higher standing (Quote 16).

### Time-poor, self-expectations, and systemic issues

Another commonly mentioned barrier to health professionals engaging with TEN was the high stress and high workload nature of healthcare. Being time-poor in general due to work and other obligations meant health professionals did not have the time to independently engage with TEN or other mental health support services (Quote 17). External stakeholders noted that being time-poor was not only mentioned as a barrier to simply engaging with TEN, but also as a barrier to self-reflection and health professionals identifying that they were experiencing mental health distress (Quote 18). Interestingly, in addition to being time-poor, the unpredictable nature of health professionals’ hours was highlighted as another issue for service adherence by internal experts (Quote 19).

The expectations that health professionals place upon themselves were also seen by both groups as a barrier to seeking help. High achieving was thought of as integral to an individual becoming a health professional, but associated beliefs and expectations of high performance could act as a barrier where health professionals may feel like they are above needing support (Quote 20). Similarly, despite being experienced in mental health management, health professionals had unrealistic optimism about the impact of workplace stress on their mental health which was seen as delaying help-seeking (Quote 21). Similarly, akin to themes around mental health stigma, health professionals placed substantial expectations upon themselves that they should be able to stoically cope with the stress of healthcare, leading to avoiding self-monitoring and help-seeking (Quote 22). Interestingly, the role of health professionals as care-providers with expertise in managing mental health was seen as somewhat paradoxical to this lack of self-care and help-seeking.

The cause for some of these self-expectations was placed on systemic issues; however, the impact of systemic issues extended beyond self-expectations to other barriers to service implementation. Internal experts reported there was a perception among health professionals that mental health services were not resolving the systemic issues, such as long hours and workplace culture, that primarily contributed to health professionals’ mental health distress (Quote 23). Without changing these systemic factors, individual support was seen as little more than a short-term solution. Instead, there was a view that interventions and support needed to be led by regulatory bodies and organisations to address the systemic causes of poor mental health among health professionals.

### Mental health stigma and mandatory reporting

The most mentioned barrier to health professionals engaging with TEN and mental health services was mental health stigma within healthcare. Health professionals were viewed as being reluctant to seek help, discuss mental health with their colleagues or superiors, or even take steps to look after themselves, due to stigma towards mental health in the healthcare profession. This stigma was commonly associated with concerns around mandatory reporting and how this may affect either their career progression or professional registration (Quote 24). This concern went beyond avoiding broaching mental health distress to colleagues, to avoiding seeking and accessing treatment that would be listed in a health professional’s medical record for fear that this may also be used against them (Quote 25). Indeed, even talking to a mental health professional was commonly seen as opening oneself up to the risk of mandatory reporting. Given how central mandatory reporting was as a barrier to help-seeking among health professionals, TEN stakeholders highlighted the efforts of both TEN and their organisations to clarify the rules around mandatory reporting. Interestingly, internal experts saw this stigma was seen as being driven by senior health professionals, who were believed to think that admitting to mental health distress indicated a lack of suitability for healthcare (Quote 26). Overtime, junior staff who survived within this environment would then come to embody this stigma because they themselves experienced it.

Another contributing factor to mental health stigma was health professionals’ role as caregivers, and how help-seeking was seen as either incongruous with this role, or that it diminished the health professional’s standing as a caregiver (Quote 27). Similarly, in terms of self-care, a culture of presenteeism (i.e., attending work even when unwell) was viewed as contributing to stigma around taking leave from work to avoid burnout or reduce mental health distress. This culture emphasised not wanting to impact colleagues’ workloads or to be seen as not taking on their share of the workload (Quote 28).

### Lack of awareness, information overload, and too much noise

Another key theme that emerged from external stakeholders as a barrier to health professionals engaging with TEN was a general lack of awareness about TEN among its target audience. This problem was viewed as being compounded by the myriad existing mental health support services and how TEN differentiates itself, as well as the sheer volume of communications health professionals already receive. Initially, from their work engaging with their respective audiences and promoting TEN, external stakeholders frequently noted a lack of awareness of TEN (Quote 29). A problem often mentioned as contributing to this lack of awareness was the volume of information health professionals receive, primarily through email. This meant that information about TEN, whether it came from external stakeholders’ organisations or healthcare employers, was frequently missed (Quote 30). Indeed, even once health professionals were aware of TEN, a further challenge was how TEN could differentiate itself enough from the other similar mental health support services to justify a health professional engaging with it. While external stakeholders acknowledged that TEN did have advantages over these other services, engaging with health professionals to explain this was typically a laboursome task.

### Competition

External stakeholders commonly highlighted Employee Assistance Programs (EAPs) as both the main competition and as a barrier to specific employers promoting TEN to their employees. EAPs are provided voluntarily by employers where employees can access confidential support for personal matters that may impact their workplace performance. The fact that an employer may be already funding a service that provided similar support to TEN was thought to act as a barrier (Quote 31). While, as discussed above, external stakeholders viewed TEN as having advantages over EAPs, they did acknowledge that these services do have some advantages of their own, such as greater flexibility with use at work (Quote 32). Despite this, stakeholders did highlight that TEN’s national scale was a positive and that this could be used to its advantage. Existing services, including EAPs and other mental health support services, were seen as focusing on metropolitan areas, with there being less in the way of support for health professionals in regional and remote areas of Australia.

### Digital services

While external stakeholders noted TEN’s digital nature as a perceived advantage of the service, both groups also emphasised the concerns of health professionals to engage with digital services. Purely digital mental health support was perceived by external stakeholders as concerning if used as a replacement for traditional person-to-person support. Some external stakeholders also expressed concerns that to low technological literacy could be a barrier to use (Quote 33), while both groups noted concerns around effectiveness of digital services related to the unidirectional nature of digital resources and a lack of engagement compared to person-to-person care (Quote 34).

## Suggested changes to TEN and increasing service uptake

### Website navigation and service accessibility

External stakeholders also highlighted several suggested changes to both the digital and person-to-person components of TEN in order to better tailor the service to health professionals. One of the main suggestions was to improve navigation on the TEN website, both to increase ease of understanding the resources available, as well as to ensure there is a simple pathway to the person-to-person services (Quote 35). Similarly, and owing to the concerns around anonymity highlighted earlier, accessing person-to-person services without providing identifying information was also suggested as a way to reduce barriers to health professionals accessing care (ES Quote 36). Lastly, given that health professionals may not pre-emptively seek mental health support, ensuring that wait times are short for person-to-person services was considered crucial.

#### TEN as a general health professional mental health support service

Another theme from external stakeholders was the balance between TEN as a support service for all health professionals and TEN being tailored for different health professionals. External stakeholders emphasised that different health professionals face different problems, and that this lack of specialisation may hinder engagement with TEN from each group (Quote 37). In terms of improving engagement, external stakeholders viewed a more specialised service as promoting recognition for each profession and ensuring the service is appealing and perceived as useful. The nature and degree of this specialisation varied somewhat; however, with some external stakeholders indicating that while specialisation is generally wanted it may not always be that necessary. Indeed, a more practical option may be to keep the digital components as broad, while ensuring that person-to-person services provide a more tailored approach (Quote 38).

### Severity of mental health

Another theme that emerged from external stakeholders in relation to changing TEN and improving service usage was the language around mental health. There was a general view that an emphasis on more severe mental health distress may be off-putting to health professionals or not relevant to a broader audience (Quote 39). Interestingly, external stakeholders instead suggested emphasising burnout in advertisements and resources, as this terminology was seen as more acceptable and appealing to health professionals.

### Peer endorsement

While a range of implementation strategies were suggested by both external stakeholders and internal experts, by far the most suggested related to peer endorsement. The best and most reliable form of peer endorsement was recommendations from colleagues or other health professionals, which was seen as the most trustworthy source (Quote 40). Despite the emphasis on peer endorsement, both groups understood the difficulty in generating this type of endorsement. Instead, other avenues were suggested to either generate peer endorsement or act as a surrogate, such as referral from healthcare providers, engagement with “champions” within workplaces, attending medical conferences, and case studies.

## Discussion

The COVID-19 pandemic amplified the risk of poor mental health among health professionals (Greenberg et al., 2020; Smallwood et al., 2021). To respond to this impending crisis, Black Dog Institute (BDI) and the Australian Federal Department of Health created a digital-first mental health service – The Essential Network (TEN) – open to all Australian health professionals (Baldwin et al., 2022). As part of an evaluation of TEN (Coleshill et al., 2022), we conducted and coded semi-structured interviews about TEN with two expert groups: representatives from Australia’s peak health professional and mental health bodies and researchers and related roles from within our institute with expertise in health professionals’ mental health. The resulting analysis offered wide-ranging and nuanced perspectives on TEN’s strengths, opportunities for improvement, and the workplace culture alongside which TEN operates.

TEN was viewed as having several advantages for engaging health professionals with a mental health service both in general and in comparison with existing services. Broadly, these related to TEN being a freely available blended care service – with the digital component allowing for greater accessibility and anonymity, as well as TEN being provided by BDI – an organisation distinct from health professionals’ existing networks. With respect to our service structure, digital treatment components were praised for offering privacy and round-the-clock access. TEN’s digital hub was viewed positively as a centralised repository of mental health resources, unlike others which some participants described as having haphazardly proliferated to saturation during the pandemic. However, our participants were clear that digital resources should form part, but not all, of a mental health service for health professionals. Some participants were concerned that digital-only services might not be effective and would lack the therapeutic connection of person-to-person treatments. TEN’s blended care approach was praised as a desirable hybrid of digital and person-to-person, allowing what many saw as a self-directed stepped-care service, in which health professionals could choose from a range of care levels depending on their needs.

Taken together, these perspectives can guide those designing services for health professionals on how to blend digital and person-to-person therapies in a way that respects consumer autonomy, ensures digital tools focus on resources that are likely to be needed 24/7, and keeps human connection available. Internal Experts suggested addressing scepticism about the effectiveness of digital therapeutics, as within such a self-serve model there will undoubtedly be consumers who can benefit from a digital-only approach, but who may need reassurance of this approach being worth their limited time. All participants made clear the importance of high-quality digital user experience (UX)/user interface (UI), with the current site criticised for poor navigability due to being a subsite within the larger BDI site.

Beyond digital, our participants believed that clinical (i.e., person-to-person) services for health professionals should offer low- or no-cost services that are independent from employer-provided services, local health services, and public health schemes where mental health consultations may appear on health records to which colleagues and employers could be privy. Such independence seemed of particular importance in rural areas, where anonymity was likely impossible if only local services were available. These opinions echo a common refrain across the broader health professional literature: privacy and confidentiality are critical elements of any mental health service built for health professionals (Clement et al., 2015; Edwards & Crisp, 2017; Zaman et al., 2022). While all mental health services should be designed with privacy and confidentiality in mind, health professionals may need highly detailed privacy and confidentiality information to feel safe.

From our participants’ point of view, the promotion of mental health services also required a nuanced approach. Industry representatives spoke of the overwhelming volume of communication about mental health supports over the past two years, making it hard for TEN to establish awareness and communicate its point of difference. Beyond awareness, most stakeholders saw Employee Assistance Programs (EAPs) as TEN’s primary competition, especially where employers were unaware of TEN’s distinguishing features, such national availability or being exclusive to health professionals. Peer endorsement was the most recommended communication channel, albeit with acknowledgement of the accompanying ethical and logistical challenges. Destigmatised language was seen as essential when promoting services to health professionals, with “burnout” a much-preferred term over “mental illness” or “psychological distress”.

Sadly, and largely mirroring existing research (Essex et al., 2023; Ledingham et al., 2019), many participants described entrenched barriers to care for health professionals. Representatives from professional organisations highlighted how health professionals’ high health literacy could affect how they select and engage with services, potentially holding resources and practitioners to higher scrutiny than a typical mental health consumer or being only willing to access services from practitioners with specific levels of training and experience. Participants also discussed the frenetic pace of healthcare and a culture of self-reliance as barriers to engagement with TEN, advising that some health professionals may be too busy to take stock of their mental health needs. Somewhat poignantly, participants suggested that health professionals may be better at recognising the need for mental health care in others than in themselves. All participants spoke of how health professionals may perceive cultural pressure to cope independently, viewing help-seeking as something appropriate for patients, but not practitioners. Together, these factors highlight the difficulty in implementing support services among health professionals, as they are aware of the factors that contribute to poor mental health and symptoms, but often fail to (or prefer not to) recognise it in themselves.

Shame apparently remains the greatest barrier to health professionals getting the mental health care they deserve. The concerns about privacy and confidentiality mentioned above appear to stem from fears about the interpersonal consequences of seeking mental health support, many of which seem justifiable. The stakeholder interviewees spoke of fears about being stigmatised by colleagues and superiors, along with the ever-present fear of being reported to regulatory bodies and the effect this could take on one’s career – a concern echoed by other research in Australia examining barriers to help-seeking among health professionals (Edwards & Crisp, 2017; Muhamad Ramzi et al., 2021; Wijeratne et al., 2021). Taking time off for mental health was described as potentially breaching the cultural expectation that health professionals should work as long and hard as necessary to avoid burdening patients and co-workers. While the literature on presenteeism in healthcare is mixed due to heterogeneity in design and measurement (Lui et al., 2018), this culture is likely to increase burnout, reduce productivity, and place patients at risk (Homrich et al., 2020). Self-stigma was also referenced, with several interviewees describing how some health professionals view help-seeking as making them unworthy to provide care. A related theme hovered above all our discussions: the tyranny of workplace culture in the health system. Across all participants and topics our interviewees described a damaging combination of unrealistic expectations and psychological threat, all brewing within impoverished hospitals and clinics. Stakeholders and researchers alike pointed to system reform and substantial organisational change as some of the most important contributions we could make to the mental health of health professionals.

While the participants in this study have significant expertise on health professionals’ needs as health industry representatives or researchers with experience in health professionals’ mental health, it remains that an understanding of health professionals’ needs is best answered by health professionals themselves. While such industry engagement was integral to the early development of TEN (Baldwin et al., 2022), the design of future services should consider updated industry engagement to ensure new and emerging needs have been addressed.

The interviews detailed in this paper reflect the knowledge and wisdom of health industry representatives and researchers on how mental health services such as TEN could be optimised to ensure health professionals have access to safe, effective mental health care. While digital tools were viewed as useful, most considered them a complement to much-needed clinical services that offer human connection. These services must be built around an understanding of the unique challenges of healthcare and promoted carefully, with respect for health professionals’ training and sensitivity to mental health stigma. Those designing services for health professionals will need to reconcile the tension between the high desire for personalisation and the need for large-scale implementation to ensure all health professionals who need support can access it. Health professionals may struggle to access care for many reasons, chief among them the mental health stigma and shame endemic in a health profession exhausted by years of under-investment in healthcare. By building mental health services that reflect the wisdom of our participants, we may be able to stem the rising tide of mental distress in our health professionals.

## Data Availability

The participants of this study did not give written consent for their data to be shared publicly, so due to the sensitive nature of the research supporting data is not available.

## Open scholarship

This article has earned the Center for Open Science badges for Open Materials and Preregistered. The materials are openly accessible at https://osf.io/hv9ta.
